# An intention-oriented multi-agent dialogue system for patient-centered decision-making

**DOI:** 10.1186/s12911-026-03498-x

**Published:** 2026-05-26

**Authors:** Pengfei Liu, Liang Xiao

**Affiliations:** School of Computer Science, Hubei University of Technology, Wuhan, Hubei China

**Keywords:** Multi-agent systems, Medical dialogue systems, Patient-centered decision making, Intent recognition, Interpretable reasoning

## Abstract

**Background:**

Conversational agents are increasingly used in healthcare, but most systems are designed for clinicians and insufficiently support patient-centered decision-making. Limited patient understanding of medical decisions may reduce adherence and lead to doctor-patient conflicts. Existing dialogue systems either lack interpretability or suffer from rigid architectures and limited scalability. This study aims to develop an interpretable, patient-centered medical dialogue system to improve communication and decision support.

**Methods:**

We propose an intention-oriented multi-agent dialogue system consisting of an intention understanding agent, an argument reasoning agent, and a template-based interaction agent. A medical knowledge ontology was constructed using a hybrid expert-data-driven approach, and a knowledge base was built from recent biomedical literature. The system performs intent recognition, entity extraction, and argument-based reasoning using weighted clinical evidence. A Graves’ eye disease dialogue platform was implemented to evaluate system performance through objective metrics and clinician-based user studies.

**Results:**

The system achieved an average F1 score of 85% for intent recognition and over 95% response accuracy in evaluations by physicians. User experience assessments showed performance comparable to physician-led online systems and superior to traditional medical Q&A systems. In comparative experiments, the proposed system significantly outperformed ChatGPT in personalized reasoning and interpretability across multiple evaluation dimensions.

**Conclusions:**

The proposed multi-agent dialogue system improves patient-centered communication by providing interpretable and personalized decision support. It demonstrates strong potential to enhance healthcare dialogue systems and assist clinical decision-making.

## Background

With the rapid development of Natural Language Processing (NLP) technology in healthcare, conversational agent systems have gained widespread popularity due to their interactivity and convenience. These systems play an important role in reducing the pressure on physicians in diagnosis and treatment, disease management, and health education. Conversational agents have demonstrated significant potential across diverse healthcare applications [1–6], including systems for sexuality education [7] and diabetes care (e.g., AMANDA) [8], as well as supporting lifestyle interventions such as alcohol cessation [9], smoking cessation [10], and weight loss [11]. However, existing systems primarily serve healthcare professionals and neglect the actual patient experience of care. Patients’ inadequate understanding of medical decisions may lead to non-compliance with medical advice and even trigger doctor-patient conflicts.

Conversational systems can be categorized into several types based on their design and functionality. According to [12], dialog systems can be categorized into finite state systems, frame-based systems and agent-based systems. Finite state dialog systems guide the user through a series of predefined steps or states, these systems have a low error rate but are less flexible as they cannot handle inputs outside the predefined range [13, 14]. Frame-based dialog systems, on the other hand, support more flexible user input, allow the user to drive the dialog process, and are able to proactively obtain needed information during the dialog [15–17]. Agent-based dialog systems are capable of handling more complex and diverse user inputs and communicating with external applications, but require a large corpus for training [18–20].

In order to enhance the communication between doctors and patients, better meet the knowledge acquisition needs of patients, and promote decision sharing and understanding, it is necessary to develop a medical question answering (Q&A) dialogue system. Currently, the main approaches to building dialog systems in healthcare include end-to-end and pipeline approaches. [21]. The end-to-end approach generates responses directly but lacks interpretability [22–26], while the pipeline-based architecture of the dialog system requires designing, training, and evaluating each module separately, which is not only time-consuming and labor-intensive, but also may lead to error propagation and amplification due to inter-module dependencies [27–29]. According to [21], the pipeline approach usually decomposes the dialog system into four modules: natural language understanding (NLU), dialog state tracking (DST), dialog policy (Policy) and natural language generation (NLG). The end-to-end approach is realized by a single model that directly processes natural language input and generates responses, which lacks interpretability [12]. Most of the existing dialogue systems are single-agent dialog systems [30], which lack a certain flexibility and scalability.Current dialog systems mostly use a single agent architecture, which limits their flexibility and scalability [30].

For this reason, this paper proposes a medical dialog system based on multi-agent technology. Multi-agent technology has the following characteristics: 1) Autonomy and distribution: each agent has independence in the system, and is able to perceive and make decisions autonomously, thus demonstrating higher adaptability and flexibility in complex and changing environments. 2) Interaction and communication: agents are able to exchange information and share knowledge among themselves, and reach common goals through coordinated actions, which is essential to meeting the diversified needs of users. 3) Division of labor and cooperation: Agents work together to solve complex problems through clear division of labor and effective cooperation, utilizing their respective professional skills and roles, reflecting the efficient coordination of collective intelligence in problem solving [30]. The multi-agent system is divided based on the idea of a single duty, including an intent understanding agent, an argument reasoning agent, and a template-based interaction agent. We define the functional modules within each agent and design a mechanism for communication and cooperation among the multi-agents, which enables the agents to share dialog information and medical knowledge with each other so as to effectively answer multiple questions from patients.

In a multi-agent dialog system, cooperation between agents is built on shared domain knowledge. To facilitate cooperation between agents, this paper utilizes an ontology to represent concepts and relationships between concepts in the medical domain. The ontology is reasonable and can be applied to a variety of question and answer tasks in a dialog system. The argument reasoning agent constructs a medical knowledge base based on the ontology by extracting clinical evidence from the PubMed literature library. Utilizing the information sharing mechanism between agents, the intent understanding agent obtains prior knowledge of the domain to automate the construction of a domain terminology lexicon and an intent training corpus, thus possessing the capability of intent understanding. The template-based interaction agents, on the other hand, generate dialogues by guiding or combining data and knowledge through dialogues.

To validate the effectiveness of the multi-agent system architecture, we constructed the Graves’ Eye Disease dialog system based on the proposed system architecture. We constructed an intent test set, and the results showed that the dialog system achieved an average F1 score of 85% on the intent recognition task. In addition, 15 physicians from partner hospitals tested the system, and the system’s question and answer accuracy exceeded 95%. The study also addressed nine patient experience assessment dimensions, comparing the multi-agent system, an online physician Q&A system, and a traditional medical encyclopedia Q&A system through a questionnaire. The results show that the multi-agent system is effective in enhancing patient experience and can provide personalized healthcare services close to the level of professional doctors. Compared with ChatGPT, a current popular large language model, our system performs better in personalized reasoning and interpretability of dialogues, and is more adaptable to the needs of medical Q&A tasks.

The main work of this paper includes the following 3 points:

- **Medical knowledge ontology**: in this paper, we propose a medical knowledge ontology that is capable of modeling complex medical concepts and relationships in the medical literature with reasoning and completeness, and can be applied to multiple Q&A scenarios in the medical domain.
- **Multi-agent system architecture** : this paper defines the functional modules of each agent and designs the interaction and cooperation mechanism between agents, so that each agent can make full use of the dialogue information and knowledge to complete the dialogue task more efficiently.
- **Graves’ eye disease dialogue platform** : the platform is built on a multi-agent dialog system that provides accurate and interpretable dialogs based on debated reasoning with patient information, and supports 15 intent types of dialogs, covering most of the issues faced by patients during treatment.

The rest of the paper is structured as follows: Section “Methods” introduces the medical knowledge ontology and describes the architecture of the multi-agent system; section “Results” provides the experimental results; section “Discussion” demonstrates two case studies of the dialog system; and, finally, section “Conclusions” concludes the full paper and proposes future research directions.

## Methods

### Ontology

Medical knowledge is usually derived from semi-structured and unstructured texts, such as medical literature and clinical guidelines. These textual information cannot be directly recognized and utilized by computers. Ontologies provide a rich and expressive data model that captures various real-world relationships between entities, such as inheritance, union, and functions. For this reason, this paper uses ontologies to capture heterogeneous clinical knowledge from multiple sources.

Currently, the construction of ontologies includes 3 main approaches: expert-driven methods, automated data-driven and hybrid methods. Expert-driven methods construct ontologies through the expertise of domain experts, ensuring accuracy and depth, but relying on subjective judgment. Automated data-driven approaches utilize technological means to automatically infer concepts and their attributes, which is efficient and scalable, but may lack in flexibility and depth. Hybrid approaches combine the strengths of both approaches by automating the generation of ontology frameworks, which are then refined by experts to improve comprehensiveness and accuracy.

**Fig. 1:**
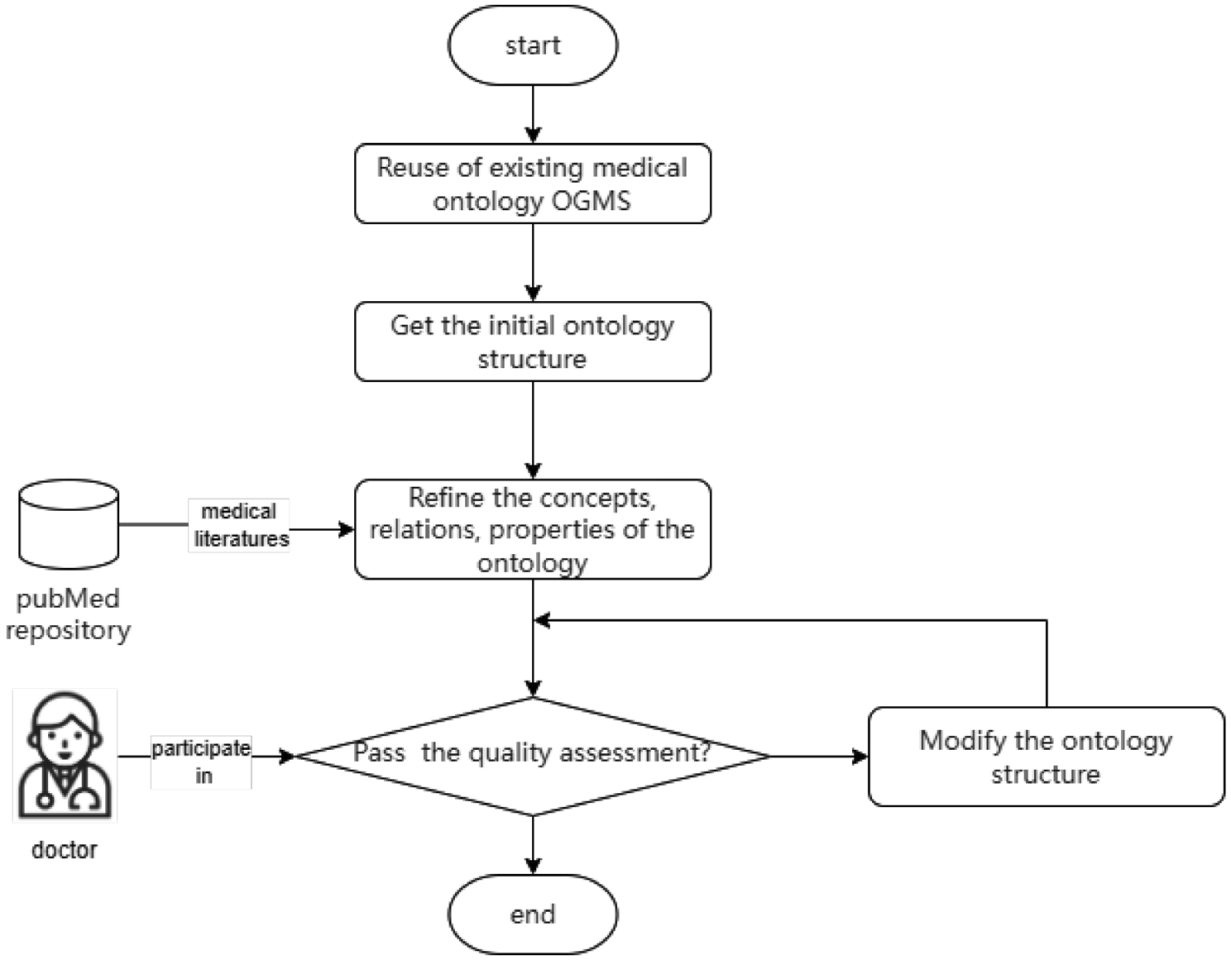
The flow chart of the development of the ontology

In this paper, a hybrid approach is used to build the ontology, and the construction process is shown in Fig. 1. In order to improve the efficiency of ontology development and the reusability of the ontology, firstly, the available medical concepts such as symptom, treatment, etc. are filtered from the existing medical ontology OGMS(Ontology of General Medical Science) [31]. Then, the concepts, relationships and attributes in the ontology are refined by mining richer semantic information from medical literature through data-driven approaches. Then, the ontology is quality audited with the help of domain experts’ expertise, and finally the medical knowledge ontology is constructed. The domain experts here come from the doctors cooperating with a hospital, and through their help, a complete, consistent and reusable ontology is constructed in this paper.

**Fig. 2:**
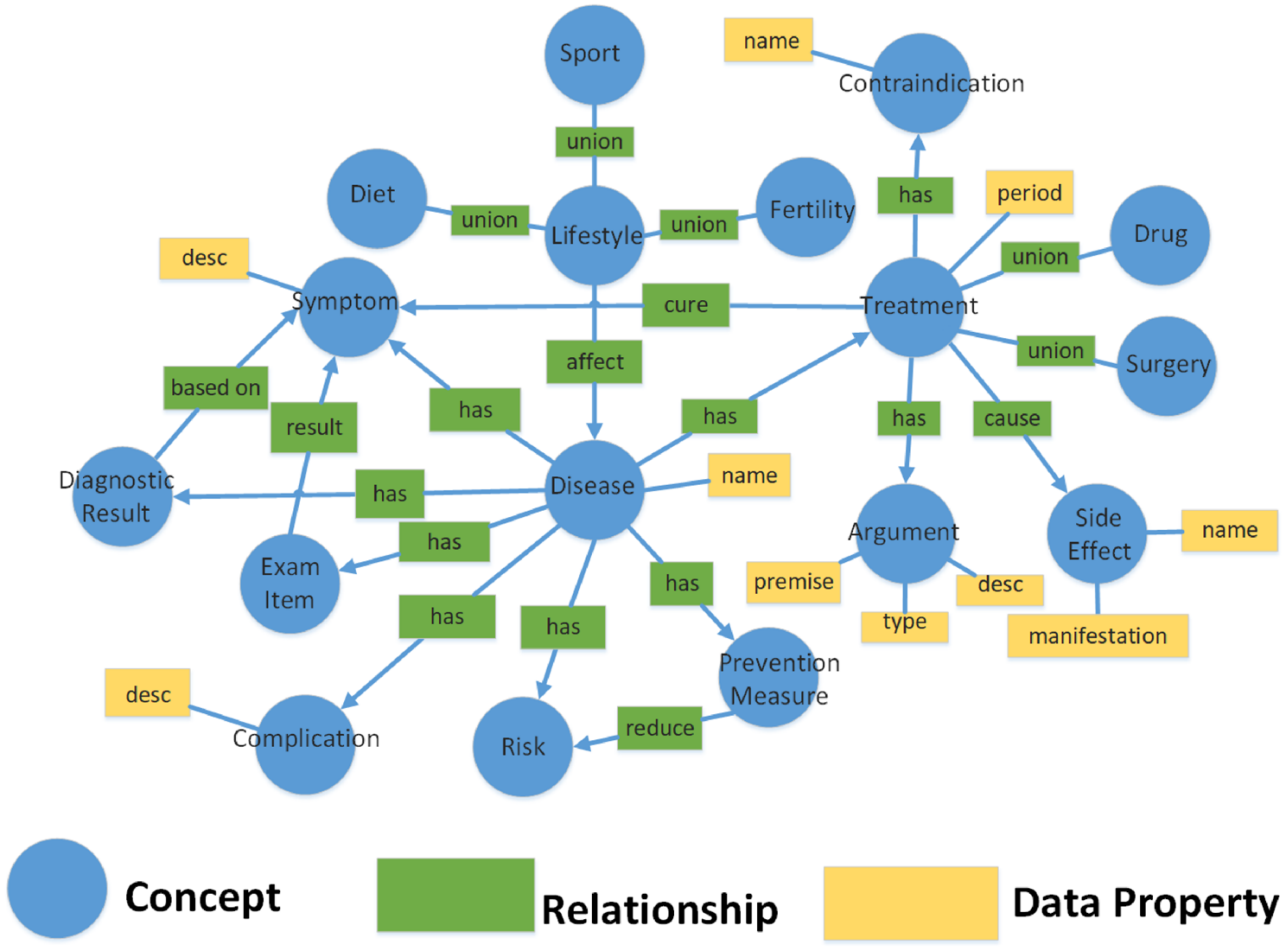
Medical knowledge ontology fragments

In this paper, the ontology schema layer construction was accomplished using Protege software, which supports the OWL ontology language [32]. The segments in the medical knowledge ontology constructed in this paper are shown in Fig. 2, where concepts are represented using circles, relationships are represented using arrows, and attributes of concepts are represented using rectangles. The ontology determines the range of questions that the dialog system can answer, and if the knowledge involved in a question is not defined by the ontology, the dialog system will not be able to respond. Ontology is mainly used in the field of postoperative Q&A, capturing clinical evidence from clinical guidelines with reasoning capabilities for tasks such as alternative treatment options, interpretation and risk warning. Ontology is central to intent understanding and argumentative reasoning in multi-agent dialog systems, and we describe the connection between ontology and multi-agents in detail in Section “Results”.

### System architecture

**Fig. 3:**
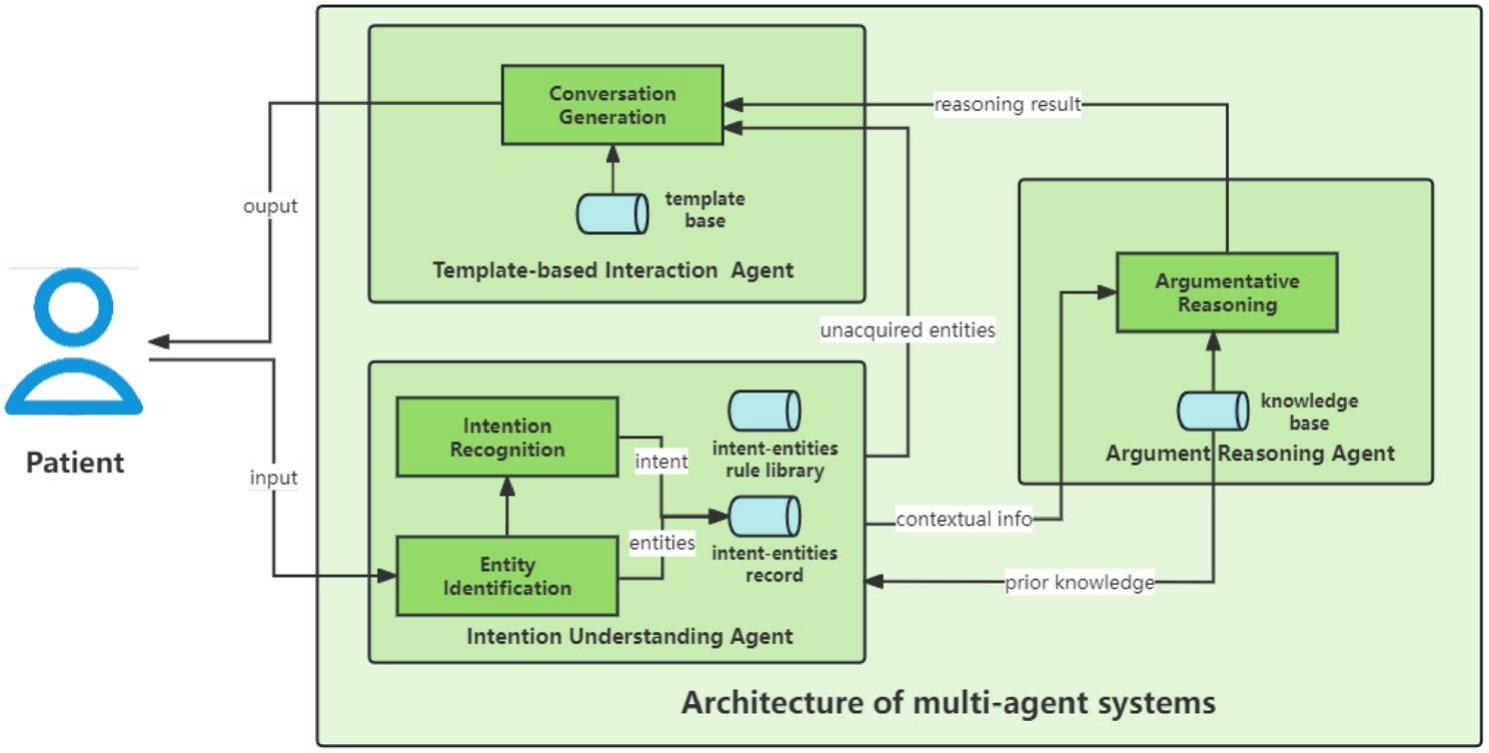
Architecture of a multi-agent system

In a multi-agent dialog system in the healthcare domain, as shown in Fig. 3, the system consists of three parts: an intent understanding agent, a argument reasoning agent, and a template-based interaction agent. Each of these agents assumes an independent function, while working together to advance the dialog through collaborative operations. The Intent Understanding Agent understands the user’s intent based on user input and stores important dialog information. The argument reasoning agent acquires dialog context information and performs debate reasoning based on the knowledge base to process the user’s intent. The template-based interaction agent interacts directly with the user and uses the template library to generate dialog content combining user data and clinical knowledge to generate dialog.

Each agent focuses on a specific task, which not only follows the design principles of multi-agent systems, but also helps to improve the maintainability, scalability and flexibility of the system. Next, the functionality of these agents and the specific implementation of their internal components are discussed in depth.

### Intention understanding agent

The core task of an intent understanding agent in a healthcare dialog system is to accurately identify user intentions and their associated entities. Intentions are the goals or behaviors that the user expects to achieve, while entities are the key information needed to realize those intentions. The accuracy of intent understanding is key to the effectiveness of a dialog system; incorrect identification may result in the system providing irrelevant feedback and affecting user satisfaction.

Currently, intent recognition techniques are categorized into three main types: rule-driven, machine learning, and deep learning. Rule-driven approaches rely on expert-defined rules that are easy to understand and implement, but have limited scalability. Comparatively, machine learning and deep learning methods are able to automatically extract recognition rules by learning a large amount of labeled data, reducing human intervention and improving recognition accuracy, but these methods require a large amount of high-quality training data.

**Fig. 4:**
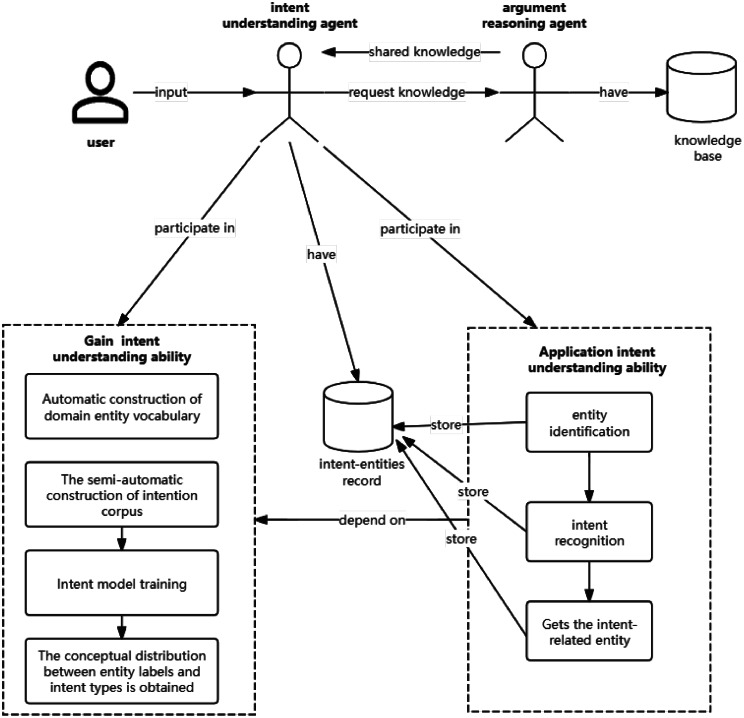
A flowchart for obtaining and applying intent understanding capabilities through information sharing between agents

In this paper, we semi-automatically construct an intent corpus through multi-agent inter-agent communication and collaboration, combined with ontology structure (section “Ontology”) and instantiated knowledge base (to be introduced in section “Argument reasoning agent”). This approach not only improves the utilization efficiency of domain knowledge, but also reduces the bias between intent recognition and knowledge base reasoning, which helps to construct intent understanding models quickly and efficiently. Figure 4 illustrates how an intent understanding agent can utilize the shared cooperation mechanism to acquire and apply intent understanding capabilities.

#### The acquisition of the ability to understand intent

The functions of an intent understanding agent in a healthcare dialog system cover two main areas: entity recognition and intent categorization. Entity recognition aims to extract key domain concepts from user input. In this paper, we achieve this goal by constructing a vocabulary and applying vocabulary matching techniques.

The process of constructing a domain vocabulary is briefly described as follows:1) the intentional understanding agent sends a request to obtain domain expertise from the argument reasoning agent. 2) The intention understanding agent obtains concepts and examples from the domain, constructs an initial vocabulary, and expands the vocabulary with the Free Thesaurus Thesaurus.

Free Thesaurus is an online thesaurus containing over 145,800 unique entries. Table 1 shows some examples of terminology dictionaries.

**Table 1:** Examples of term dictionary

Entitiy	Examples
Symptom	redness of eyelids,pain when gazing,swelling of eyelids
Complication	hypertension,diabetes,hyperthyroidism
Drug	teprotumumab,prednisolone,methylprednisolone
Surgery	orbital decompression, squint / lid surgery,radiotherapy
Risk	deterioration risk,occurrence risk,progression risk

In this paper, we adopt a multi-agent communication and cooperation mechanism to semi-automatically construct an intent corpus in the following steps:1) Determine the types of intents based on the concepts and their relationships in the ontology.2) Design a matching pattern for each intent, covering entity concepts, attributes, relationships and leading statements.3) Instantiate the matching patterns using the concepts, relations and attributes in the knowledge base to form the intent corpus.

**Fig. 5:**
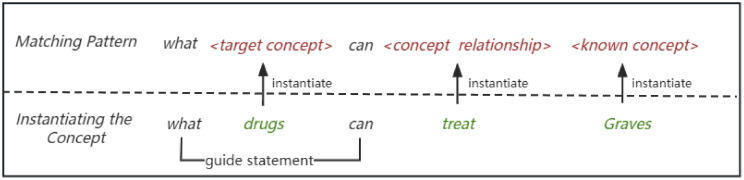
Example of a matching pattern for a concept relationship

**Fig. 6:**
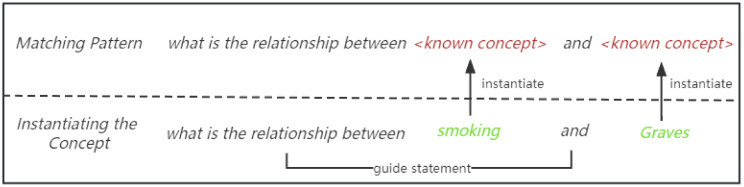
Example of a matching pattern to find a target concept

**Fig. 7:**
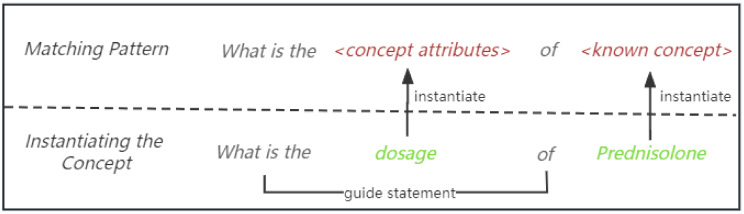
Example of a matching pattern for a concept property

Intention matching patterns are mainly categorized into three types: patterns that ask for a relationship between two known concepts (e.g., Figure 5), patterns that ask for a known concept and a relationship corresponding to another concept (e.g., Figure 6), and patterns that ask for attributes within a concept class (e.g., Figure 7). The corpus for training the intent model is generated by continuously instantiating these patterns.

Table 2 lists the 15 types of intentions that can be recognized by the intention understanding agent. Since the entity types in each type of intention are relatively independent, this paper uses the plain Bayesian algorithm for categorization. The probability formulas for entity categories and intent types are as follows:

$$p(x|c)=\frac{count(x,c) +k}{N_c +K*N}$$

- $count(x,c)$ denotes the number of times feature $x$ appears in the training data of category $c$.
- $N_c$ denotes the total number of times category $c$ is in the training data.
- $$N $$ denotes the total number of different features in the training data.
- $k$ is a smoothing parameter, usually taken as 1, which is used to deal with data sparsity and ensure that the conditional probability is not zero.

**Table 2:** Types of intentions

Type of Intent	Explanation	Example
Disease_Prevention	Ask about preventive measures for the disease	How to prevent disease recurrence?
Disease_Symptom	Ask about disease symptoms	What are the usual symptoms of Graves’ eye disease?
Disease_Clinical_Examinations	Inquire about what Clinical Examinations are required for diagnosing the disease.	What Clinical Examinations are needed to diagnose Graves’ disease?
Diseases_TreatmentOptions	Seeking ways to treat the disease	What treatments are available for Graves?
TreatmentEffect_Complaints	Users are less satisfied with the treatment results.	I’ve been on medication for 3 months now, and I’m a little disappointed that the recovery is not too obvious.
TreatmentOption_SideEffects	Ask about the side effects of your treatment plan.	What are the side effects of radiation therapy for hyperthyroid eye disease?
TreatmentOption_Lifestyle	Ask about the impact of the treatment plan on lifestyle.	Does the drug Sage have an effect on pregnancy preparation?
Drug_Usage	Ask how the medication is used	What is the dosage and duration of the medication
Treatment_Effectiveness	Ask about the effectiveness of the treatment plan	Is radiation therapy the best treatment for thyroid eye disease.
Replace_TreatmentPlan	Alternative treatment options	Why can’t radiation therapy be used instead of drug therapy?
Lifestyle_Impacts	Impact of lifestyle on disease	Can smoking and drinking lead to worsening of the disease?
Disease_AdvocacySports	Exercises promoted by the disease	What activities should I do to speed up my recovery from hyperthyroidism?
Disease_ProhibitedSports	Sports prohibited by the disease	What activities are not allowed with eye diseases?
Disease_AdvocatedFoods	Foods promoted by the disease	What to eat for faster recovery from eye disease?
Disease_ProhibitedFoods	Foods prohibited by the disease	Do you need to avoid spicy and sour foods for hyperthyroidism?

This smoothing technique effectively avoids the zero probability problem and enhances the stability and generalization ability of the model.

#### Application of the ability to understand intent

In the medical dialog system, the intention recognition ability of the intention understanding agent is its core function. The process is shown on the right side of Fig. 4 and consists of the following four steps:

(1) Entity recognition: extract key entities from user input to generate labeled sentences.

(2) Intent classification: determine the specific intent of the user.

(3) Obtaining intent-related entities: obtain entities directly related to the user’s intent.

(4) Information storage: save the intent type and entity information for use in multiple rounds of dialog.

The steps of entity recognition are as follows: 1) Segmentation processing: the input sentences are first segmented to generate a list of words. 2) Lexical matching: the list is then traversed and each word is compared with the labeled words in the lexical database. 3) Labeling entities: words that are successfully matched are labeled with the corresponding entity category; words that are not matched are labeled with non-entities. 4) Outputting the results: finally, the algorithm will output the sentences with lexical annotations that provide the necessary detailed information for subsequent intent recognition.

The pseudo-code for entity recognition is shown in Algorithm 1.

**Figure:**
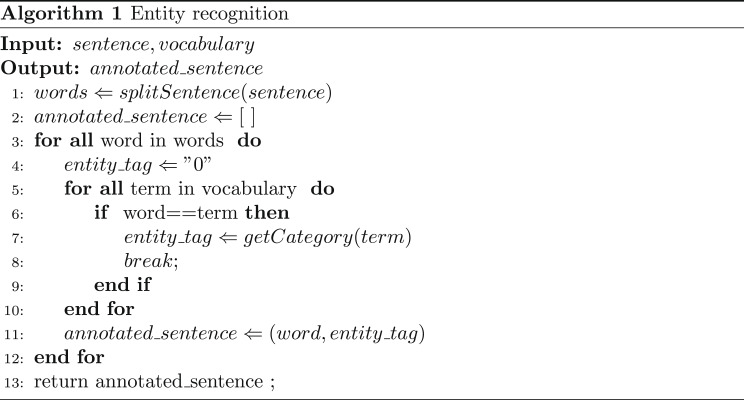

The intent classification process relies on the results of the previous step of entity recognition and consists of the following three steps:1) Preprocessing and lexical annotation: first, the user input is processed using Algorithm 1 to obtain utterances with lexical annotations. Each utterance consists of a series of words labeled with lexical properties.2) Probability computation: second, based on the lexical annotations obtained in the first step, the association probabilities between each lexical annotation and each intent type are computed and these probabilities are accumulated to form the total probability for each intent type. These accumulated probabilities will be stored in an array of intent probabilities.3) Intent recognition: finally, the intent type with the highest probability is selected from the intent probability array as the final intent recognition result.

The pseudo-code for intent classification is shown in Algorithm 2.

**Figure:**
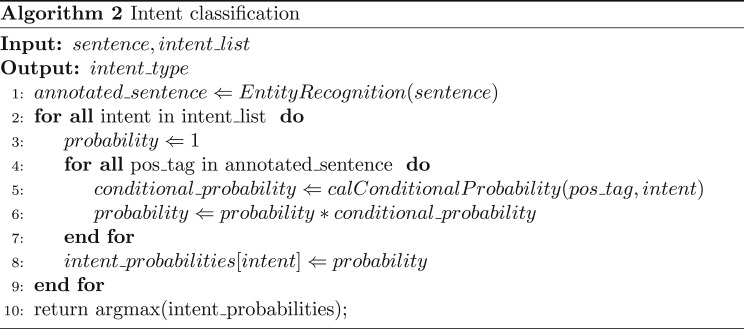

Once the intent understanding agent recognizes the user’s intent, it then needs to identify the intent-related entity information, which are the key information needed by the dialog system to process the user’s intent. For example when a user asks about the side effects of a drug, then the name of the drug needs to be recognized so that the system can access the side effects of the drug.

Intention-related entity information is divided into two categories: mandatory entities and optional entities as shown in Table 3. Mandatory entities are the information that must be acquired to accomplish intent recognition, while optional entities are not required but can help the dialog system to provide a more personalized dialog. If the user question does not contain all mandatory entities, the template-based interaction agent needs to guide the user to add relevant information.

All the entity categories entered by the user have already been recognized in step 1, so it is only necessary to filter the entity information corresponding to the intent types according to the structure defined in Table 3. Finally, the obtained intent types and entity information are stored for subsequent debating reasoning and multiple rounds of interactive dialog.

**Table 3:** Intent-related entities

Type of Intent	Mandatory Entities	Optional Entities
Disease_Prevention	disease name	risk type
Disease_Symptom	disease name	null
Disease_Clinical_Examinations	disease name	null
Diseases_TreatmentOptions	diagnostic result, treatment option name	preference
TreatmentEffect_Complaints	disease name,intervention,treatment time	reason
TreatmentOption_SideEffects	treatment option name	null
TreatmentOption_Lifestyle	treatment option name,lifestyle name	null
Drug_Usage	drug name	time, frequency, dose
Treatment_Effectiveness	treatment option name	treatment time
Replace_TreatmentPlan	diagnostic result,intervention, desired treatment option,complications	reasons
Lifestyle_Impacts	disease name,lifestyle name	null
Disease_AdvocacySports	disease name	null
Disease_ProhibitedSports	disease name	null
Disease_AdvocatedFoods	disease name	null
Disease_ProhibitedFoods	disease name	null

### Argument reasoning agent

The core function of a argument reasoning agent is to acquire knowledge from external data sources, store it in a knowledge base, and utilize that knowledge base for logical reasoning. The knowledge base not only contains rich medical knowledge that provides the basis for logical reasoning, but also directly affects the breadth and depth of the questions answered by the dialog system.

#### Construction of the knowledge base

The source of knowledge was mainly from the open medical literature of PubMed, and the literature was acquired by manual screening. Considering the rapid updating of medical knowledge, this paper only includes literature from the last three years and excludes some literature with low impact factor to ensure the quality of the literature base. The medical literature covers four application scenarios: prevention, diagnosis, treatment and rehabilitation.

**Fig. 8:**
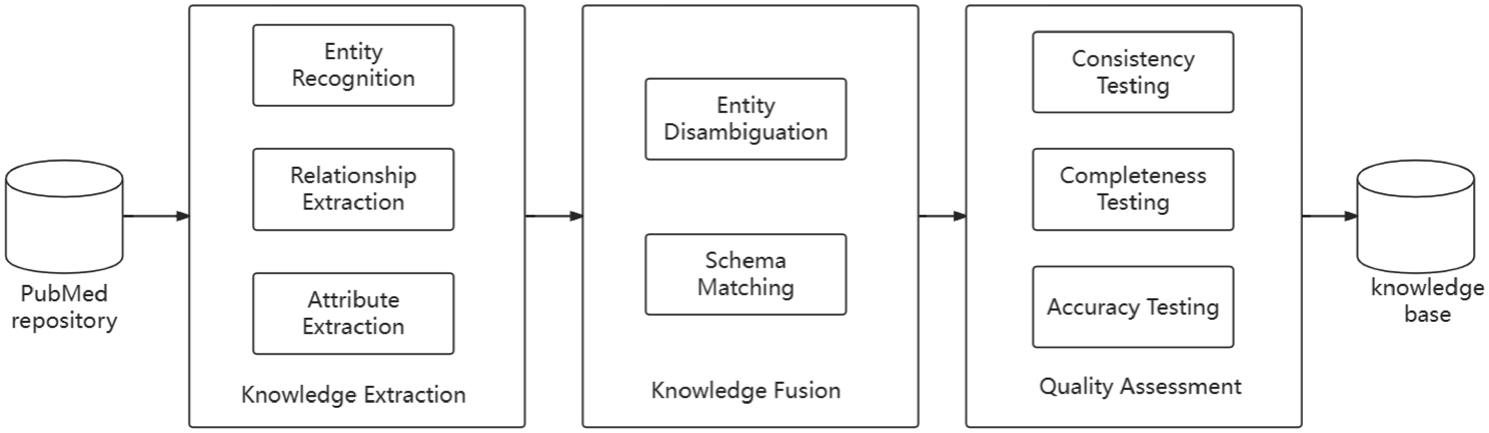
Knowledge base construction flowchart

This paper adopts a top-down approach to construct a knowledge graph, covering three key steps: knowledge extraction, knowledge fusion and quality assessment,as shown in Fig. 8. The specific processes are as follows:1) Knowledge Extraction: Entities, relationships and attributes are extracted from medical literature using SemRep tool to form knowledge triples. This step may produce duplicate and ambiguous triples. 2) Knowledge Fusion: Medical terms are annotated through UMLS(Unified Medical Language System) medical vocabulary to eliminate duplicate data and link the triples to the ontology layer to complete knowledge fusion. 3) Quality Assessment: The generated knowledge is assessed for completeness, consistency and accuracy to ensure the quality of the knowledge graph.

**Fig. 9:**
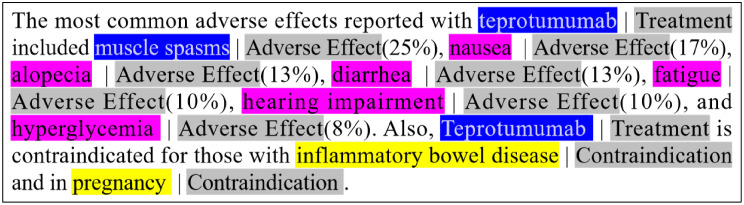
Example of SemRep tool parsing results

SemRep, short for Semantic Representation, is a rule-based natural language processing tool. Based on the standardized medical concepts, concept types and semantic relationships between concepts in UMLS, it extracts “subject-predicate-object” triples from natural language texts. As shown in Figure 9, the SemRep tool is able to recognize the conceptual categories of entities in the literature, where the vertical line “$|$” is preceded by the entity and the vertical line “$|$” is followed by the category of the entity. Semantic recognition of the text by the SemRep tool is able to generate knowledge triples for both treatment-cause-sideeffect and treatment-has-contraindication categories.

**Fig. 10:**
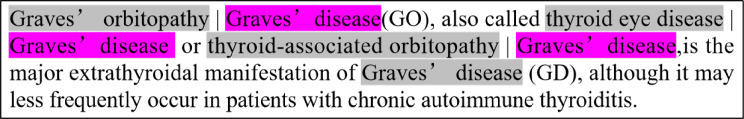
Examples of standardization of terminology

The process of knowledge extraction generates a large number of knowledge triples, which may have duplicate or ambiguous anomalies and need to be further fused to improve the accuracy and usability of the knowledge graph. In this paper, UMLS is used to annotate the terms so as to solve the ambiguity problem. For duplicate triples, they are removed. Figure 10 illustrates the process of UMLS to standardize different expressions of the same entity concept into the same term.

In order to verify the quality of the knowledge graph, this paper carries out visual analysis, semantic query and other operations on the constructed knowledge graph to check the consistency, completeness and accuracy of the knowledge, and this process is mainly done manually.

The argument reasoning agent is capable of automatically loading relevant knowledge and performing logical reasoning to process user intent. The workflow of the argument reasoning agent includes the following four steps:1) Obtaining the dialog context, including the intent type and entity information, from the intent understanding agent. 2) Retrieve knowledge related to the current dialog context in the knowledge base.3) Perform logical reasoning using the retrieved knowledge and context information.4) Passes the reasoning results to the interaction agent for further processing.The following will detail how a argument reasoning agent reasons.

#### Argument-based interpretable reasoning

**Fig. 11:**
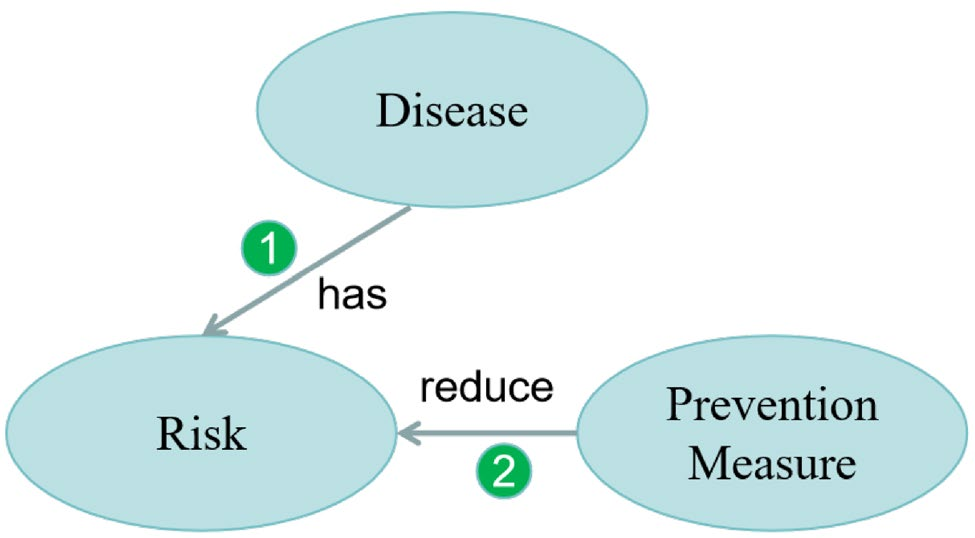
Two-hop query with the intent type of Disease_Prevention

Most of the intents can be answered with only a single or multi-hop query by the agent based on the knowledge. For example, for the “Disease_Symptom” intent type, the agent directly obtains entities whose nodes are of type Symptoms from the knowledge base. For the “Lifestyle_Impact” intent type, it first matches the lifestyle and disease nodes and then returns the relationship between them. Figure 11 shows a two-hop query for the “Disease_ Prevention” intent type: first identifying the disease risk factors and then determining the corresponding preventive measures.

However, querying from the knowledge base alone is not sufficient to answer all types of intents. For example, for the two intent types “Disease_TreatmentOptions” and “Replace_TreatmentPlan”, the agent needs to extract the arguments for the treatment plans from the knowledge base and comprehensively evaluate the feasibility of each treatment plan based on the type of argument (for or against). In order to realize computable debate reasoning, this paper classifies the strength of evidence recommendation into eight types based on the semantic information of verbs or adjectives in the medical literature, as shown in Table 4. For example, when there is a prohibition or contraindication in the clinical evidence, the type of the evidence is exclusion.

**Table 4:** Types of arguments and calculable values

Argument Type	Numeric
for	2
moderately for	4
strongly for	6
confirm	8
oppose	− 2
moderately oppose	−4
strongly oppose	−6
exclude	−8

Algorithm 3 describes the argument-based interpretable reasoning process of the argument reasoning agent. The inputs include intent, entity information, knowledge base and thresholds, and the outputs are dialog types and reasoning results. The steps of the algorithm are as follows:1) Identify all relevant treatment options from the knowledge base based on intent and entity information and initialize the weights.2) Traverse all relevant evidence under each treatment plan, match patient data to determine the validity of the evidence, and recalculate the weights based on the type of evidence.3) Obtain the weights, supporting evidence, and opposing evidence for each treatment plan.4)Answer the intent based on the results of the reasoning. For the intent of type of treatment option, select the option with a weight greater than the threshold; for the intent of alternative treatment plan, compare the magnitude of the weights of the two treatment options.

**Figure:**
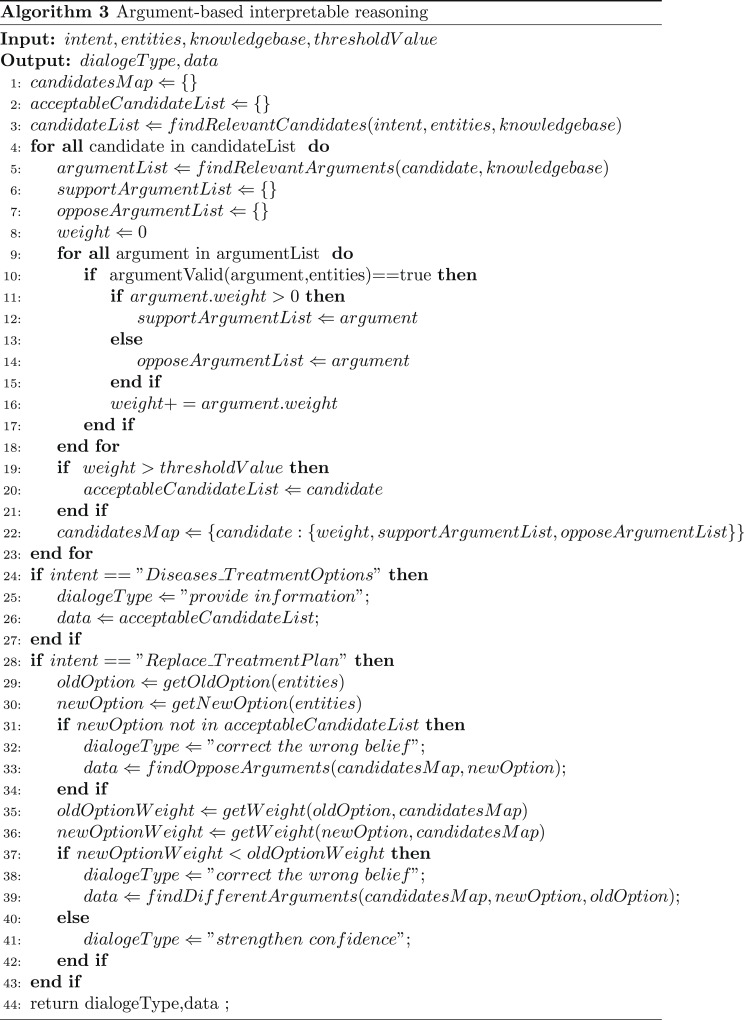

### Template-based interaction agent

The interaction agent is responsible for interacting with the user in a dialog, generating knowledge-based and process-guided dialogs. Knowledge-based dialogs are generated by combining dialog templates and reasoning results from the argument reasoning agent. Process-guided dialogs, on the other hand, cover the initiation, interruption, termination, and information about the guiding entity of the dialog, and do not rely on the argument reasoning agent.

Table 5 shows the dialog templates for process guidance, while Table 6 lists the dialog templates used to answer the user’s intent. These templates consist of fixed statements and variables, which include the patient’s health data and medical knowledge. The dialog templates are designed to reduce the workload of the interacting agents and increase the response speed.

**Table 5:** Templates for dialogue guidance

Conversation Guide Type	Template Example
dialogue start	hello.I’m Ice, the medical assistant, how can I help you?
conversation break	are you still there? If there are no other questions, we will end the conversation.
end of conversation	you are welcome, feel free to ask me questions!
entity guidance	what is the {Entity Name}?

**Table 6:** Templates for each intention

Type of Intent	Template Example
Disease_Prevention	{Disease} prevention measures include {Preventive Measure1}, {Preventive Measure2} …
Disease_Symptom	Common symptoms of {Disease} include {Symptom1},{Symptom2} …
Disease_Clinical_Examinations	The following clinical examinations are usually required for disease: {Exam1}, {Exam2} …
Diseases_TreatmentOptions	In your case, {Treatment Option} is suitable for you.
TreatmentEffect_Complaints	The treatment cycle for {Disease} is long and slow, and the effect is usually noticeable in the second half of the treatment. Please maintain patience and good mood, which also has a certain effect on the recovery of the disease.
TreatmentOptions_SideEffects	{Treatment Option} may have the following side effects: {Side Effect1},{Side Effect2} …. If you feel serious discomfort, please stop taking the medication immediately and go to the hospital for relevant examination.
TreatmentOption_Lifestyle	{Lifestyle} is prohibited/possible during the {Treatment Option}.
Drug_Usage	The instructions for taking the {Drug} are as follows: {Times} per day, {Number of Tablets} per time, before/after a meal, for a period of {Cycles}.
Treatment_Effectiveness	The {Treatment Plan} was developed in accordance with clinical practice guidelines. The {Treatment Option} is valid as indicated by {Evidence} in the clinical guidelines: having a {Diagnosis} takes precedence over the {Treatment Option}.
Replace_TreatmentPlan	{Treatment Option 1} is feasible/not feasible in place of {Treatment option 2}. For your current condition, according to the {evidence} in the clinical guidelines: having {Symptoms} is for/against {Treatment Option1}.
Lifestyle_ Impacts	{Lifestyle} is beneficial/bad for {Disease}.
Disease_AdvocacySports	The following exercises can be performed: {Exercise1},{Exercise2} to speed up recovery from the {Disease}.
Disease_ProhibitedSports	The following forms of exercise are prohibited for a confirmed {Disease}: {Exercise1},{Exercise2} …
Disease_AdvocatedFoods	Diet can eat {Food1}{Food2} … to supplement nutrition and energy to speed up the recovery of the disease.
Disease_ProhibitedFoods	Avoid the following food items: {Food1}, {Food2} …

The workflow between multiple agents from user input to system input is as follows:1) The user submits a question through the interaction interface and the message is forwarded to the Intent Understanding Agent. 2) The intent understanding agent recognizes the user’s intent, extracts the relevant entity information, and records it in the session repository. If there is unrecognized required entity information, the interaction agent generates a bootstrap statement to obtain this information. Once all the required information is collected, the process moves to the next step.3) The argument reasoning agent loads relevant knowledge from the knowledge base based on the intent and entity information and performs logical reasoning and sends the result to the interacting agent.4) The interaction agent selects the appropriate template based on the reasoning results and generates the dialog content.

## Results

In this paper, a medical dialog platform for Graves’ eye disease is constructed using the multi-agent system architecture proposed in section “Methods”. The main reason for choosing Graves’ eye disease is that the disease has a long treatment period, there may be some adverse reactions and the risk of deterioration during the treatment process, and the disease is not widely known. In this study, the system performance was comprehensively evaluated from both objective and subjective dimensions. The objective evaluation metrics include the F1 score for intent recognition and the accuracy and usage rate of dialog generation, while the subjective evaluation measures the user experience of using the system mainly by means of questionnaires.

### Intent recognition performance test

Intent recognition performance testing aims to evaluate the accuracy and performance of intent recognition models. In order to closely match the real input scenarios, this study collects patient questions from the online Q & A website “Seek Medical Help”^1^, labels their intent types, and constructs a test set. In order to ensure the reliability of the test results, the data distribution was balanced by discarding the part with too many intent types and adding the ones with fewer intent types, so that the number of each intent type was kept at about 50.

The test set is fed into the intent model, and four results are obtained by comparing the actual output intent type ×with the expected intent type y: TP (True Positive), FP (False Positive), TN (True Negative), and FN (False Negative).

The TP prediction is positive and correct; the FP prediction is positive and incorrect; the TN prediction is negative and correct; and the FN prediction is negative and incorrect;

In this paper, the performance of the model is evaluated using the F1 score, which is calculated using the following formula:

$$\begin{aligned}F1-Score&=\frac{2TP}{2TP+FP+FN}\cr&=2* \frac{Precision*Recall}{Precision+Recall}\end{aligned}$$

The formula combines Precision and Recall to provide a comprehensive metric to measure the accuracy and comprehensiveness of the model in positive class prediction. According to the data in Table 7, the average F1 score for intent recognition reaches 85%, indicating the effectiveness of the automatic construction of the intent corpus and the plain Bayesian algorithm proposed in this paper for the intent recognition method.

**Table 7:** F1 score for each type of intention

Type of Intent	F1
Disease_Prevention	83%
Disease_Symptom	84%
Disease_Clinical_Examinations	86%
Diseases_TreatmentOptions	82%
TreatmentOption_SideEffects	93%
TreatmentEffect_Complaints	84%
TreatmentOption_Lifestyle	88%
Drug_Usage	75%
Treatment_ Effectiveness	91%
Replace_ TreatmentPlan	94%
Lifestyle_Impacts	85%
Disease_AdvocacySports	83%
Disease_ ProhibitedSports	84%
Disease_AdvocatedFoods	89%
Disease_ProhibitedFoods	85%

Lower F1 scores for certain intent types may be caused by training data that does not cover the linguistic habits of a wide range of users due to insufficient feature extraction or data volume, reasons. For this reason, subsequent optimization of the intent model will optimize the intent recognition model by ensuring professionalism and consistency in data annotation, expanding the dataset through data enhancement or external data sources, and other efforts.

### User test

In evaluating the Graves’ Eye Dialog system, we first deployed it on a server and invited 15 physicians from our partner hospitals to participate in the test. Before starting to use the system, we described in detail the basics of Graves’ disease and the scenarios in which the system would be used, and asked the physicians to enter as many types of questions as possible during testing to simulate possible patient inputs in the clinic.

In the process of using the system, the user can evaluate each response by clicking the thumb icon of “up” or “down”, where “up” indicates satisfaction and “down” indicates dissatisfaction. The correct response rate and usage rate of each intent were calculated by counting the number of interactions and negative feedback of each intent. The formula is as follows:

$$Accuracy=\frac{\begin{aligned}&total\;interactions \;\cr&-\; negative\;feedback\end{aligned}}{total\; interactions}$$

The formula for the probability of intent usage is as follows:

$$Usage=\frac{\begin{aligned}&The\; number\; of\;interactions\cr&\;for\;an\;intent \;type\end{aligned}}{total\; interactions}$$

Based on the data in Table 8, we found that users are most concerned about the treatment phase, especially the issue of side effects of drugs. This reflects that patients have doubts about the advice provided by doctors or the system. Meanwhile, 8% of users expressed dissatisfaction with the treatment effect, which suggests that we need to enhance the emotional calming function of the system. In addition, the low percentage of inquiries about the lifestyle of the disease suggests the need to enhance the knowledge of postoperative life guidance.

**Table 8:** Distribution of usage and accuracy of intention types

Type of Intent	Usage	Accuracy
TreatmentOption_SideEffects	16%	97.6%
Diseases_TreatmentOptions	12%	98.4%
Drug_Usage	11%	96.8%
Treatment_Effectiveness	9%	96.3%
TreatmentEffect_Complaints	8%	95.0%
Disease_Symptom	7%	96.3%
Replace_TreatmentPlan	7%	96.4%
Disease_Prevention	6%	97.3%
Disease_ProhibitedSports	5%	96.1%
Lifestyle_Impacts	5%	99.1%
Disease_AdvocacySports	4%	97.4%
Disease_AdvocatedFoods	4%	97.6%
Disease_ProhibitedFoods	3%	97.4%
Disease_Clinical_Examinations	2%	98.3%

The system’s correct response rate for all types of intentions exceeded 95%, which demonstrated the efficient capability of the multi-agent dialog system in intention recognition.

However, there are limitations in assessing the accuracy of the system’s response only through the negative feedback button. Users tend to express dissatisfaction only when the system makes serious errors.

In order to further assess patients’ experiences with the multi-agent dialog system, a questionnaire was designed and implemented in this study. During the questionnaire construction process, this study referred to the assessment metrics used in the existing literature and incorporated the relevance of the application domains, including the assessment metrics commonly used in the application domains such as teleconsultation [33, 34], virtual clinics [35], and chronic disease management applications [36], and excluded metrics that were not relevant to the objectives of this study, such as the accessibility of monitoring devices [37]. Ultimately, the questionnaire assessed the patient experience in terms of the following nine dimensions: ease of use of the system, timeliness of communication, quality of communication, quality of care, reliability, patient preference, emotional experience, user engagement, and overall satisfaction.

Participants were asked to rate three different online medical Q&A systems, including online physician-based Q&A systems (e.g., Good Doctor^2^, Family Doctor^3^, etc.),traditional medical encyclopedic Q&A systems (e.g., Ask Me Anything^4^, etc.), and the multi-agent dialog system proposed in this paper. Online physician-based Q&A systems usually require users to make an appointment and pay a fee to get a doctor’s consultation, whereas traditional medical encyclopedia Q&A systems require users to manually type in questions and filter the answers themselves.The rating scale was on a scale of 1 to 10. A total of 15 valid questionnaires were collected in this study, and all participants were physicians. In terms of data processing, for the same assessment dimension of the same type of system, we calculated the average of all scores to derive the statistical results, which are detailed in Table 9.

**Table 9:** Average patient experience scores for online doctor based Q&A system,traditional medical encyclopedia Q&A system, and multi-agent dialog system

Assessment Dimension (1–10 points)	Online Doctor Based Q&A System	Traditional Medical Encyclopedia Q&A System	Multi-agent Dialog System
ease of use	8.0	6.5	8.3
timeliness of communication	8.5	6.2	9.0
quality of communication	9.1	6.8	8.8
quality of care	9.3	6.3	9.1
reliability	9.5	7.5	9.3
patient preference	9.8	5.5	9.6
emotional experience	9.7	4.8	8.5
user engagement	9.6	6.1	9.5
overall satisfaction	9.3	5.3	9.6

As can be seen in Table 9, in the dimensions of communication quality, medical quality, reliability, patient preference, and user engagement, the performance of the multi-agent dialogue system is close to that of the online doctor-based Q&A system, and far exceeds that of the traditional medical encyclopedia Q&A system, showing the ability to personalize and professionalize the service. In terms of ease of use, timeliness of communication, and overall satisfaction, the multi-agent dialog system outperforms the other two systems, which may be attributed to its convenient Q&A mode that does not require an appointment. In terms of emotional experience, although the multi-agent dialog system is ahead of the traditional encyclopedia Q&A systems, there is still room for improvement compared with the online doctor-based Q&A system. Traditional medical encyclopedia Q&A systems scored lower on several dimensions, possibly due to their lack of real-time interaction, personalized service, emotional intelligence, and reliability, which together affect user experience and satisfaction.

In conclusion, the multi-agent dialogue system developed in this study has achieved significant results in enhancing the patient experience and is able to provide personalized care close to professional healthcare services. With the continued optimization of key technologies such as emotion recognition, it is expected that the system will partially assume the functions of physicians in the future, helping to alleviate the current strain on healthcare resources.

### Comparison test

In this study, ChatGPT, a current popular chat dialog model, is selected for comparison. Based on the dialog context of user-system interaction described in section “User test”, we constructed a patient case library with the aim of comparing the functional differences between the multi-agent dialog system proposed in this paper and the ChatGPT dialog system. In constructing the patient case base, this study excluded patient conditions with a high degree of similarity and added individualized patient cases based on objective knowledge of clinical guidelines to ensure that the diversity of patient conditions and individual needs were met. The personalized patients specifically considered complications specific to thyroid eye disease, such as hyperthyroidism, and high-risk risk factors, such as hypertension. Ultimately, the constructed case library contains 32 cases, of which 16 are base cases, dealing with prevention and diagnostic issues in thyroid eye disease; 10 are co-morbid cases; and 6 take into account patient preferences, such as pregnancy. Each case consists of three parts: the patient’s clinical presentation/past medical history, the problem, and the reasoning results.

This paper compares the performance of the systems on two dimensions: personalized reasoning and interpretability capabilities. The personalized reasoning capability covers four main steps: intent recognition, entity guidance, introduction of dialog context knowledge, and debate reasoning, requiring the system to have a complete reasoning chain. Interpretability capability measures whether the system’s reasoning results are easy to understand from the perspective of dialog result presentation.

In order to test the system’s personalized reasoning and interpretability capabilities, this study invited 16 doctors to participate in the experiment, divided into two groups of 8 each. One group used the multi-agent system and the other group used ChatGPT, and both groups of physicians used the system as a clinical decision-making Q&A aid. We simulated patient inputs based on the case, and the doctors could view the patient’s inputs and the system’s outputs for the patient’s current contextual information on the web side. Doctors can indicate their support or oppose to the output of the system by ticking or crossing the box. If the doctor does not approve of the system’s output, he/she needs to give feedback to the system on why the output does not make sense or make suggestions. At the end of the experiment, physicians rated the effectiveness of the use of ChatGPT and the multi-agent system in clinical Q&A assisted decision making.

The assessment dimensions included five questions: 1) whether the patient’s personalized intention could be recognized and responded to; 2) whether the patient’s personalized data could be guided and collected; 3) whether arguments could be used rationally and reasoned accurately based on the conversation context; 4) whether the decision outcome took into account the patient’s personalized needs; and 5) whether the decision outcome was interpretable. For the same assessment dimensions of the same system, we calculated the average of all scores, and the results are detailed in Table 10.

**Table 10:** Average scores of ChatGPT and multi-agent dialogue systems on personalized reasoning and interpretability assessment questions

Survey Questions	ChatGPT	Multi-agent System
patient’s personalized intent can be recognized and responded to	6.1	9.4
patient’s personalized data can be directed and collected	6.3	9.2
is the ability to rationalize the use of arguments and reason accurately based on the context of the conversation	6.7	9.3
whether the outcome of the decision takes into account the individualized needs of the patient	5.3	9.6
whether the outcome of the decision is interpretable	6.2	9.1

Based on the evaluation scores shown in Table 10, this study compares the performance of a multi-agent dialog system with ChatGPT in clinical Q&A-assisted decision making. The results show that the multi-agent system significantly outperforms ChatGPT on several key dimensions, including: personalized intent recognition and response (9.4 vs. 6.1 points), personalized data guidance and collection (9.2 vs. 6.3 points), contextual argument use and reasoning accuracy (9.3 vs. 6.7 points), personalized needs consideration (9.6 vs. 5.3 points), and interpretability of decision outcomes (9.1 vs. 6.2 points). Further analyzing the feedback suggestions from doctors, this study finds that ChatGPT fails to effectively guide patients to input necessary data and introduce relevant knowledge for reasoning in a specific dialogue context, resulting in its limited reasoning ability in most Q&A scenarios. In contrast, the multi-agent dialogue system proposed in this paper is able to effectively guide patients to input necessary data and introduce relevant knowledge for logical reasoning based on the context, and thus performs better in terms of personalized reasoning capability.

**Fig. 12:**
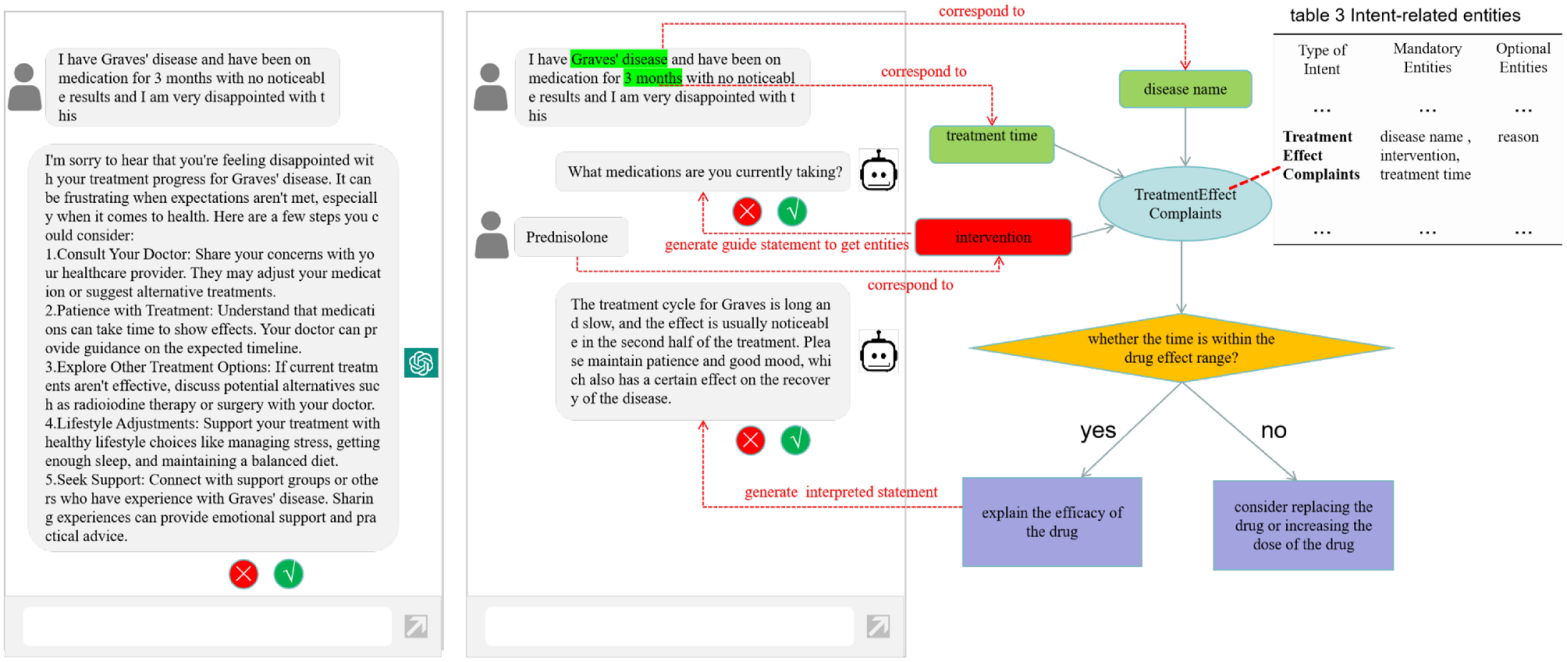
Comparison with ChatGPT - example 1

**Fig. 13:**
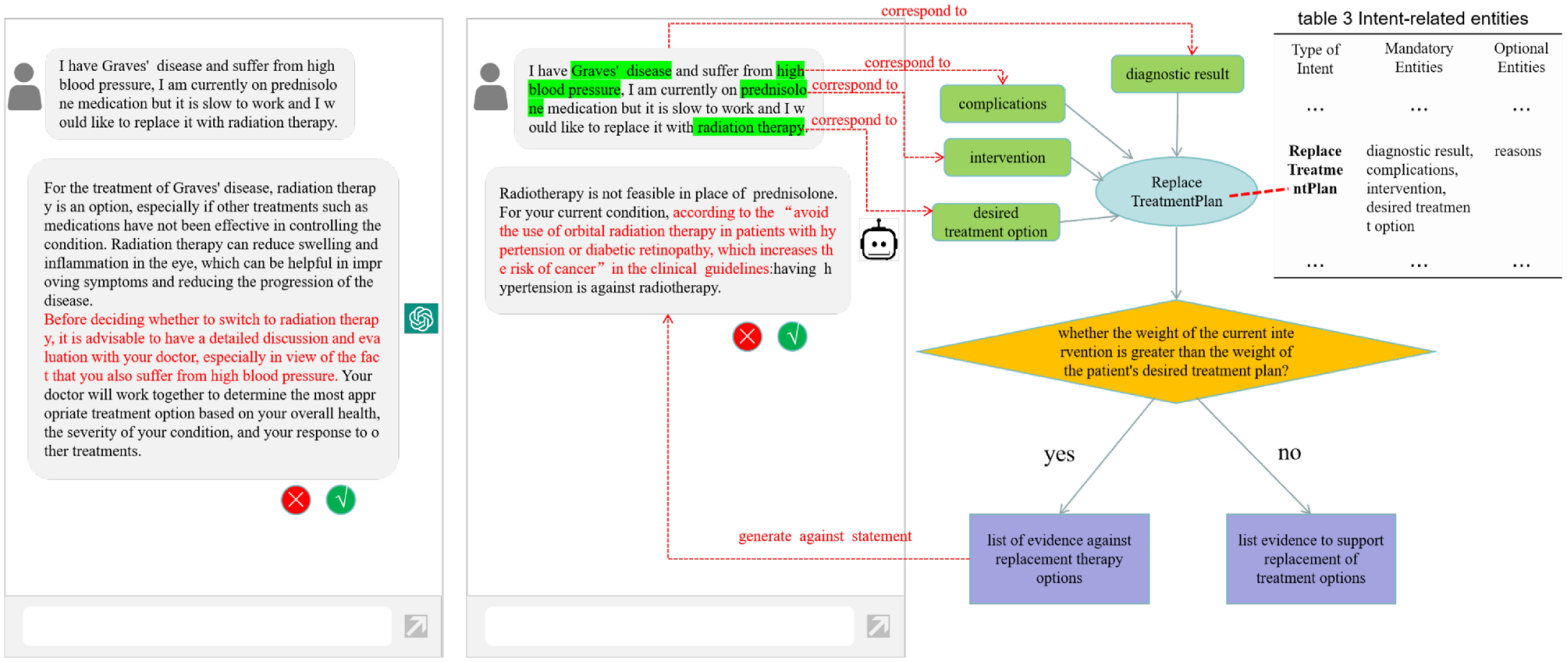
Comparison with ChatGPT - example 2

Fig. 12 and Fig. 13 show a comparison case between ChatGPT and a multi-agent dialog system in the same Q & A scenario, respectively. The left part of Fig. 12 reveals the inadequacy of ChatGPT’s ability to guide and acquire information, which limits its ability to provide personalized medical advice. In contrast, the multi-agent dialogue system proposed in this study is capable of accurately identifying the type of patient’s intention (TreatmentEffect_Complaints) and guiding the patient to provide the necessary medical information (intervention) based on the intention-related entities listed in Table 3, which enables it to provide personalized medical advice.

In the left part of Figure 13, ChatGPT fails to perform personalized reasoning when confronted with personalized patient information with more general answers. In contrast, the multi-agent dialog system in this study was able to accurately identify the intent as “Replace_TreatmentPlan”, obtain information about the entities associated with the intent (diagnostic result, complications, intervention, and desired treatment option), and reason logically based on the evidence in the clinical guidelines and the patient’s information, ultimately providing an interpretable dialog result.

In summary, the multi-agent system proposed in this paper demonstrates significant advantages in providing personalized services and enhancing decision interpretability. In contrast, ChatGPT’s performance in terms of personalized service and interpretability has room for improvement, which limits its wide application in clinical decision support. Future research will aim to validate the effectiveness and reliability of multi-agent systems in a wider range of clinical scenarios and larger user groups to ensure their broad applicability in real-world applications.

In the subsequent sections, this paper will further analyze in detail two personalized cases during user-system interactions in order to delve deeper into the advantages of multi-agent dialogue systems in terms of personalized reasoning and interpretability.

## Discussion

In this section, we demonstrate the effectiveness of our system architecture through two case studies. The patient’s condition and needs in the cases are designed based on the medical literature on Graves’ disease, while the dialog content is generated by the user interacting with the system in a set dialog context.

### Case study 1: context-aware treatment decision and interpretable reasoning

Background: Beck has Graves’ eye disease and is taking medication for it. Due to the long duration of drug treatment and the lack of significant improvement in symptoms, the current treatment plan was questioned when it was known that the relative recovered quickly after radiotherapy.Below is Beck’s conversation with the system.

**Table:** 

01 Agent:Hello.I’m Ice, the medical assistant, how can I help you?
02 Beck :I have Graves’ disease and have been on medication for 3 months with no noticeable results and I am very disappointed with this. 03 Agent:What medications are you currently taking? 04 Beck:Prednisolone 05 Agent:The treatment cycle for {graves} is long and slow, and the effect is usually noticeable in the second half of the treatment. Please maintain patience and good mood, which also has a certain effect on the recovery of the disease 06 Beck:My relative was also tested for Graves at this hospital in the first half of the year and he recovered quickly after radiotherapy. Why can’t I also have the more effective radiation therapy and instead have the drug immunotherapy which has a much slower recovery period. 07 Agent:Do you have a disease other than Graves? 08 Beck: Yes, I also suffer from high blood pressure. 09 Agent:{Radiotherapy} is not feasible in place of {Prednisolone}. For your current condition, according to the {avoid the use of orbital radiation therapy in patients with hypertension or diabetic retinopathy, which increases the risk of cancer} in the clinical guidelines:having {hypertension} is against {radiotherapy}. 10 Beck:Okay, I understand.What are the side effects of the medications I am currently taking? 11 Agent:{Prednisolone} may have the following side effects: {elevated blood pressure, blood sugar},{increased risk of infection}. If you feel serious discomfort, please stop taking the medication immediately and go to the hospital for relevant examination. 12 Beck: I would like to ask what should be taken care of in terms of lifestyle. 13 Agent:Diet can eat {protein foods},{Vitamin B foods}to supplement nutrition and energy to speed up the recovery of the disease.Avoid the following food items: {foods high in iodine}.The following forms of exercise are prohibited for a confirmed {graves}:{certain strenuous exercises}. 14 Beck:Thank you for your advice! 15 Agent:You are welcome, feel free to ask me questions!

The case involves four types of intentions: complaints about treatment effects, substitution of treatments, asking about medication side effects, and post-operative lifestyle. First, Beck complains about the ineffectiveness of the medication (line 2), which is recognized by the intent understanding agent as TreatmentEffect_Complaints.The intent understanding agent recognizes the intent’s relevant entity information based on the intent-related entities table in Table 3: Disease and Dosing Cycle, but it lacks the mandatory entity information: the name of the medication. Thus, the intent understanding agent sends the unavailable entity name, i.e., the drug name, to the template-based Interaction Agent, which in turn generates the dialogue guiding statements (line 3). After the dialog system obtains the complete intent and entity information, the argument reasoning agent first obtains the treatment period of the drug (6 months) and compares it with the patient’s medication time (3 months) to reason that the drug has a slower response. After acquiring the reasoning results, the template-based interaction agent generated a dialog (line 5) using the template library. Subsequently, based on the user input, the intent understanding agent recognizes the intent type as Replace_TreatmentPlan and reuses the entity information of the previous intent (Disease: Graves, Intervention: prednisolone) and obtains the treatment plan radiotherapy for the user’s goal from the dialog.According to Table 3, the template-based interaction agent guides the user to enter the required entity for this intention: complication (line 7). The argument reasoning agent finds the relevant arguments in radiotherapy and prednisolone from the knowledge base and uses Algorithm 3 for debate reasoning.

**Fig. 14:**
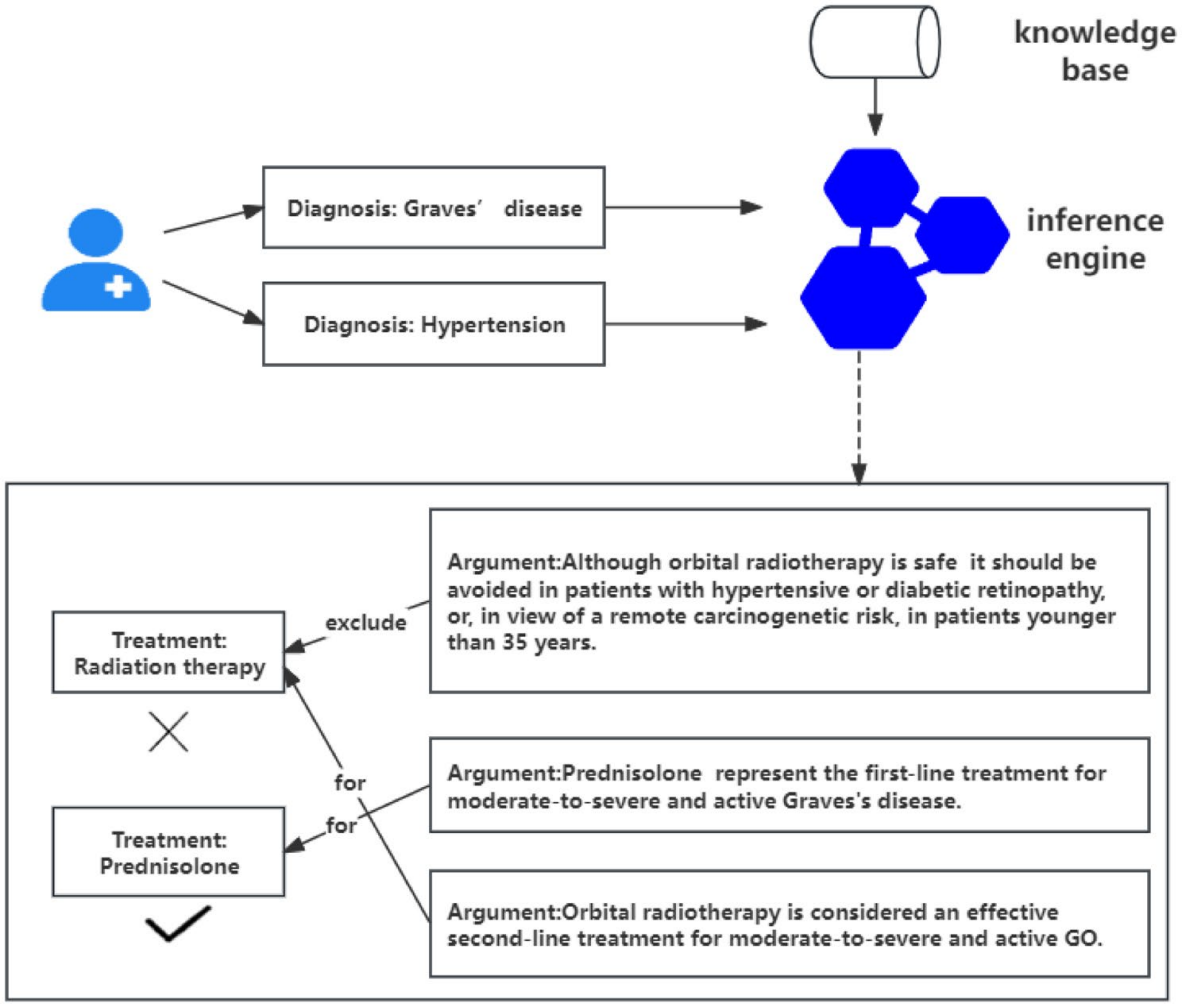
The knowledge fragment corresponding to the intention of Replace_TreatmentPlan in the case

As shown in Figure 14, the argument reasoning agent obtains a total of 3 arguments, and the radiotherapy and prednisolone treatment regimens are each supported by 1 piece of evidence of type for (weight 2), but the radiotherapy treatment regimen has an additional piece of evidence of type exclude (weight −8). Since the weight of the radiotherapy treatment regimen is less than 0 and the prednisolone treatment regimen is greater than 0, the argument reasoning agent reasons that the radiotherapy treatment regimen cannot be used for the patient. The argument reasoning agent sends the reasoning result and the evidence against radiotherapy to the template-based interactive agent. The template-based interaction agent generates a dialog using the template library based on the reasoning and knowledge of the debate reasoning agent (line 9).

In line 10, the user’s intent type is TreatmentOption_SideEffects, and the required entity information (disease name, drug name) associated with this intent type has been obtained from the previous dialog (lines 2 to 5), so the argument reasoning agent finds the side effects of the drug from the knowledge base based on the disease and drug name. The template-based interaction agent generated the dialog by matching the side effects of the drug with the variables in the template library (line 11).

In line 12, the user asks about the lifestyle of Graves’ eye disease. According to Table 4.3, the intent types Disease_AdvocacySports, Disease_ProhibitedSports, Disease_AdvocatedFoods, and Disease_ProhibitedFoods are sub-intents of the lifestyle intent type. Therefore, the argument Reasoning agent acquires the knowledge related to these 4 types of intents from the knowledge base and passes it to the Interaction agent, which generates the dialog (line 13).

**Fig. 15:**
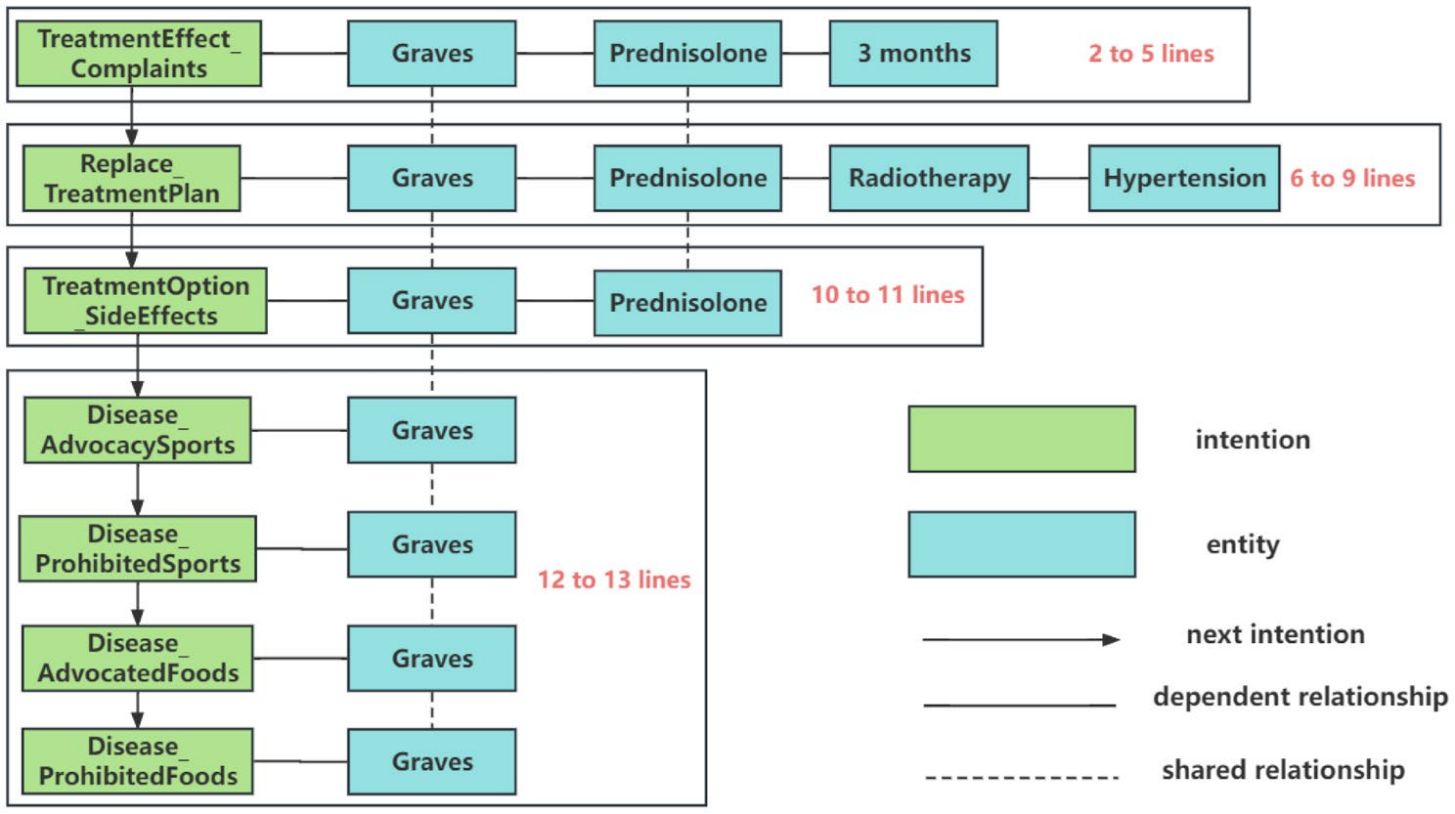
Case1 corresponding dialogue intent tree

As the dialog proceeds, the intent understanding agent continuously recognizes intent and entity information from the user input. This information constitutes the intent tree structure, which is stored in the Intent-Entity Record Repository. Figure 15 illustrates the intent tree for the case. The fact that entity information of different intent types can be used in conjunction with each other indicates that the dialog system has a certain level of contextual understanding and can effectively handle complex dialog tasks.

### Case study 2: preference-aware treatment recommendation and personalized dialogue

Background: Alice, 30 years old, has Graves’ hyperthyroidism and is currently on medication. Due to advice from her family, Alice would like to get pregnant as soon as possible, but she is concerned that her current medication may be interfering with pregnancy preparation. Below is Alice’s conversation with the system.

**Table:** 

01 Alice: I have Graves’ hyperthyroidism and I am currently taking Sage, will this medication affect my pregnancy preparation?
02 Agent: {Pregnancy} is prohibited during the {Sage}. 03 Alice: I want to prepare for pregnancy right away, can you give me a replacement drug? 04 Agent: In your case, {propylthiouracil} is suitable for you. 05 Alice: And what are the side effects of this medicine? 06 Agent: {propylthiouracil} may have the following side effects: {causing a rash},{abnormal liver function} and {causing a decrease in white blood cells}. If you feel serious discomfort, please stop taking the medication immediately and go to the hospital for relevant examination. 07 Alice: Okay, I understand. What is the dose of propylthiouracil? 08 Agent:The instructions for taking the {propylthiouracil} are as follows: {3} per day, {1} per time, after a meal, for a period of {2 months}. 09 Alice: Okay, thanks. 10 Agent: You’re welcome, are there any other questions? 11 Alice: No more for now. 12 Agent: Okay, feel free to ask any questions.

The case again involves multiple intentions. First, Alice asks if the medication she is currently taking will affect pregnancy (line 1). After learning that the medication does affect pregnancy preparation (line 2), Alice raises the need for alternative treatment options (line 3). It is important to note that, unlike Case 1, Alice is not sure which treatment options would treat Graves’ hyperthyroidism without affecting pregnancy preparation, so her intent type is a request for the system to provide appropriate treatment options.

The argument reasoning agent looks up all possible treatment options and their associated arguments based on the relevant entity information for that intent type: pregnancy preparation (preference) and Graves’ hyperthyroidism (diagnosis). Using Algorithm 3 to calculate the weight of each treatment option, propylthiouracil was ultimately determined to be the appropriate treatment choice, which not only met Alice’s preference but also effectively treated her condition. To be on the safe side, Alice asks about the side effects of the drug (line 5) and the dosage of the drug (line 7). lines 9 to 12 are knowledge-base independent dialogs, where the agent proactively asks the user if he has any other questions, and after receiving a clear no, the system calls the end-of-dialog statement, which says that the user is welcome to ask questions at any time.

## Conclusions

In this study, a medical dialog system was constructed using multi-agent technology, aiming to enhance the efficiency and quality of doctor-patient communication through an innovative system architecture. The functional modules of each agent are specified in the study, and the inter-agent interaction and cooperation mechanism is designed to enhance the flexibility and scalability of the system.

In the experimental evaluation phase, the proposed multi-agent dialog system achieved an average F1 score of 85% on the intention recognition task. The average question and answer accuracy of the system exceeds 95% through a simulation test with 15 physicians. User feedback shows that the multi-agent system is able to provide personalized medical services close to the level of professional doctors, which significantly improves the user experience. Compared with the current popular large language model ChatGPT, the multi-agent system constructed in this study has obvious advantages in terms of personalized reasoning and interpretability.

This paper further provides a detailed analysis of two dialog cases, demonstrating the ability of the multi-agent system in providing accurate and fluent dialog. Overall, this study provides a convenient and efficient new way for doctor-patient communication, which is a positive contribution to the development of medical dialog systems.

In the future, we plan to further extend the functionality of the multi-agent system. First, we will introduce sentiment analysis tools to enhance the user experience. In addition, we will enhance the input-output capabilities of the system, such as adding speech recognition. Finally, given the sensitive nature of conversations in the medical field, we will enhance privacy protection measures to ensure that the security and privacy of patient information is not compromised.

## Data Availability

The datasets generated and/or analysed during the current study are not publicly available due to institutional policies and confidentiality agreements but are available from the corresponding author on reasonable request.

## Footnotes

1 Seek Medical Help: https://www.xywy.com/

2 Good Doctor: https://www.haodf.com/

3 Family Doctor: https://www.familydoctor.com.cn

4 Ask Me Anything: https://www.120ask.com/
